# A Novel Peroxidase Mimics and Ameliorates Alzheimer’s Disease-Related Pathology and Cognitive Decline in Mice

**DOI:** 10.3390/ijms19113304

**Published:** 2018-10-24

**Authors:** Jia Xu, Kai Wang, Ye Yuan, Hui Li, Ruining Zhang, Shuwen Guan, Liping Wang

**Affiliations:** 1 School of Life Sciences, Jilin University, Changchun 130012, China; xujia16@mails.jlu.edu.cn (J.X.); wangkai17@mails.jlu.edu.cn (K.W.); yeyuan18@mails.jlu.edu.cn (Y.Y.); lihui18@mails.jlu.edu.cn (H.L.); rnzhang15@mails.jlu.edu.cn (R.Z.); guanshuwen@jlu.edu.cn (S.G.); 2 Engineering Laboratory for AIDS Vaccine, Jilin University, Changchun 130012, China; 3 Key Laboratory for Molecular Enzymology and Engineering, the Ministry of Education, Jilin Universtiy, Changchun 130012, China

**Keywords:** Alzheimer’s disease, deuterohemin-AlaHisThrValGluLys, cognitive ability, Aβ deposition, oxidative stress, pro-inflammatory cytokines

## Abstract

Alzheimer’s disease (AD) is the most common neurodegenerative disorder in the elderly, which is characterized by the accumulation of amyloid β (Aβ) plaques, oxidative stress, and neuronal loss. Therefore, clearing Aβ aggregates and reducing oxidative stress could be an effective therapeutic strategy for AD. Deuterohemin-AlaHisThrValGluLys (DhHP-6), a novel deuterohemin-containing peptide mimetic of the natural microperoxidase-11 (MP-11), shows higher antioxidant activity and stability compared to the natural microperoxidases. DhHP-6 possesses the ability of extending lifespan and alleviating paralysis in the Aβ1-42 transgenic *Caenorhabditis elegans* CL4176 model of AD, as shown in our previous study. Therefore, this study was aimed at exploring the neuroprotective effect of DhHP-6 in the APPswe/PSEN1dE9 transgenic mouse model of AD. DhHP-6 reduced the diameter and fiber structure of Aβ1-42 aggregation in vitro, as shown by dynamic light scattering and transmission electron microscope. DhHP-6 exerted its neuroprotective effect by inhibiting Aβ aggregation and plaque formation, and by reducing Aβ1-42 oligomers-induced neurotoxicity on HT22 (mouse hippocampal neuronal) and SH-SY5Y (human neuroblastoma) cells. In the AD mouse model, DhHP-6 significantly ameliorated cognitive decline and improved spatial learning ability in behavioral tests including the Morris water maze, Y-maze, novel object recognition, open field, and nest-building test. Moreover, DhHP-6 reduced the deposition of Aβ plaques in the cerebral cortex and hippocampus. More importantly, DhHP-6 restored the morphology of astrocytes and microglia, and significantly reduced the levels of pro-inflammatory cytokines. Our findings provide a basis for considering the non-toxic, peroxidase mimetic DhHP-6 as a new candidate drug against AD.

## 1. Introduction

Alzheimer’s disease (AD) is an age-related progressive neurodegenerative disorder mainly affecting people over 65 years of age. AD patients exhibit senile dementia characterized by a progressive deterioration in memory, cognition, and behavioral patterns [1,2]. The molecular basis of AD consists of a massive deposition of extracellular misfolded protein aggregates (also known as senile plaques or SP) and hyperphosphorylation of the tau protein (neurofibrillary tangles, NFT), leading to the loss of synapses and neurons, and damage to the cerebral cortex and hippocampus [3,4]. Several hypotheses have been proposed for the mechanistic basis of AD pathogenesis, and the amyloid cascade hypothesis is the most widely accepted [5]. According to this hypothesis, β-amyloid peptides (Aβ1-40 and Aβ1-42) are produced due to aberrant β and γ-secretase proteolytic cleavage of the amyloid precursor protein (APP), which results in the extraneuronal accumulation of Aβ [6,7]. These aggregates induce oxidative stress by increasing reactive oxygen species (ROS) [8] and triggering mitochondrial dysfunction, tau protein hyperphosphorylation, and neurotoxicity [9]. Persistent oxidative stress further aggravates Aβ production and aggregation and tau phosphorylation, leading to neuronal apoptosis, neuroinflammation, and metabolic disturbances [10,11]. This vicious cycle of Aβ deposition and oxidative stress is the main driver of AD progression [12]. Therefore, targeting Aβ is a promising therapeutic strategy for AD, and can be achieved in the following was: (1) reducing Aβ peptide formation by enhancing the activity of α-secretase (non-amyloidogenic pathway), and reducing that of β and γ-secretase (amyloidogenic pathway) [9], (2) inhibiting the aggregation and formation of low molecular weight Aβ1-42 oligomers, which are more neurotoxic than other forms [13], and (3) enhancing Aβ clearance to restore oxidative stress homeostasis and control inflammation [14,15].

Several β-sheet breaker polypeptides and antioxidants [16,17,18] have been tested for their Aβ-targeting abilities and their efficacy in alleviating the symptoms and progression of AD. The active decapeptide inhibitor RR (RYYAAFFARR) [19] was designed to target the critical Aβ11-23 extension by hydrophobic and electrostatic interactions and hydrogen bonding to inhibit the fibrillation of Aβ1-40. The β-sheet breaker peptide acetyl-LPFFD-amide, iAβ5p [20], and its modified versions, such as the C-terminal trehalose-conjugated Ac-LPFFD-Th [21] and VVIACLPFFD (VCD10)-AuNP [22], exert a protective effect against Aβ oligomer-induced neurotoxicity by inhibiting Aβ1-42 aggregation. The CPO-Aβ17-21P peptide [23] ameliorates AD-related cognitive decline in APPswe/PSEN1dE9 (APP/PS1) transgenic mice by inhibiting Apolipoprotein E (apoE) and Aβ binding, reducing amyloid deposition, and regulating the inflammatory response. Some anti-Alzheimer peptides have even entered the preclinical and clinical testing phases [24]. For example, Davunetide (AL-108, NAPVSIPQ) [25] is the smallest active element of the vasoactive intestinal peptide, which mediates glial cell-induced neuroprotection, has shown potent neuroprotective, memory-enhancing, and neurotrophic properties in preclinical studies, and is under clinical trial [24,26]. The neurotrophic FGL-2 (the FG loop peptide) and FGL-loop peptides [27], which are high molecular weight stimulants of fibroblast growth factor (FGF), are also currently in the early stages of clinical trials as potential AD therapeutic drugs [24]. In addition, these functional peptides are non-toxic, and are small enough to easily pass through the blood–brain barrier (BBB) [28].

Deuterohemin-AlaHisThrValGluLys (DhHP-6) is a novel peroxidase mimetic peptide that is based on the structure of the natural microperoxidase-11 (MP-11). Its catalytic site is constituted by a five-coordination iron-porphyrin ring that is occupied by an imidazolium of histidine at the second site of polypeptides, which is the same as MP-11 [29]. Compared to MP-11 [30], DhHP-6 has better water solubility and stability, and is also easier to synthesize [31]. In addition, DhHP-6 shows a strong peroxidase activity, uses hydrogen peroxide in oxidation reactions, and has an antioxidant effect on cultured rat lens crystalline, protecting it from galactose-induced cataracts [32]. Furthermore, DhHP-6 extends the lifespan of *Caenorhabditis elegans* by 19%, and improves its survival rate during acute heat stress (35 °C) and paraquat-induced acute oxidative stress, indicating the potential anti-aging effects of DhHP-6 as well [33,34]. Our previous results demonstrated that DhHP-6 significantly extended the lifespan and reduced paralysis in the Aβ1-42 transgenic *C. elegans* strain CL4176, which is a nematode model of AD.

To further study the role of DhHP-6 in AD pathogenesis and cognitive decline, we used the transgenic APPswe/PSEN1dE9 mouse model of AD. We first analyzed the interaction between DhHP-6 and Aβ, and found that this mimetic peptide significantly reduced and dissembled Aβ1-42 aggregates. In addition, DhHP-6 was non-toxic and neuroprotective in the human and murine cellular models in vitro. DhHP-6 treatment on APPswe/PSEN1dE9 mice led to a significant reduction in amyloid deposition in the cerebral cortex and hippocampus, resulting in a considerable restoration of the cognitive and learning abilities of the AD mice.

## 2. Results

### 2.1. DhHP-6 Reduces the Aggregation of Aβ1-42 and Protects Neurons from Aβ Oligomers Toxicity In Vitro

**Figure 1:**
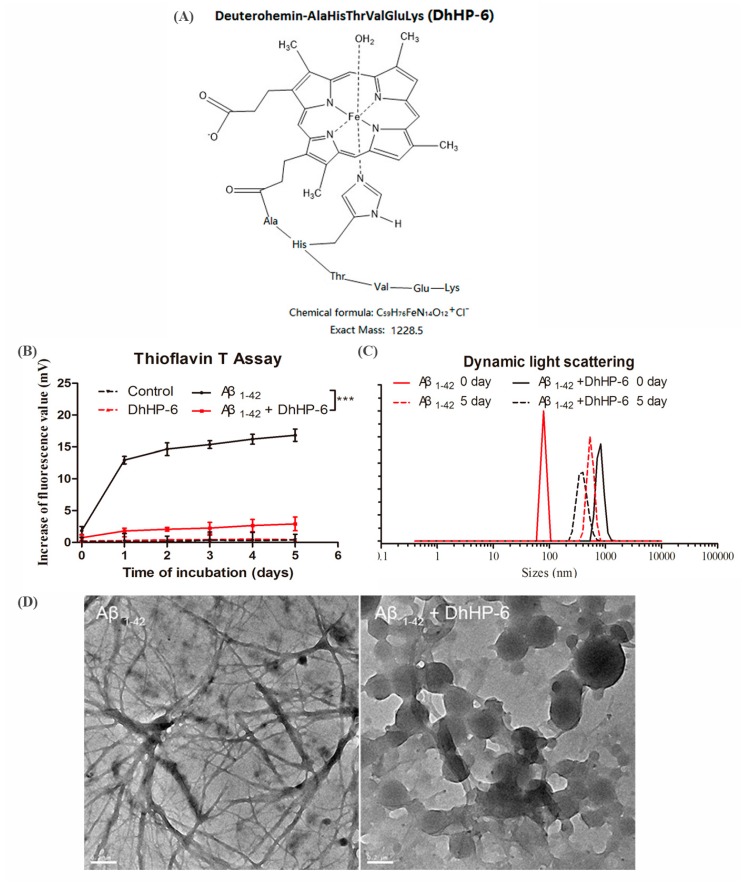
Molecular structure of deuterohemin-AlaHisThrValGluLys (DhHP-6) and its inhibitory effect on amyloid β (Aβ)1-42 aggregation in vitro. (**A**) Molecular structure of the peroxidase mimetic Deuterohemin-AlaHisThrValGluLys (DhHP-6). (**B**) Thioflavin T (Th-T) assay evaluated the aggregation of Aβ1-42 (10 μM, black line) and Aβ1-42 incubated with DhHP-6 (10 μM:10 μM molar ratio, red line) at 37 °C for five days (*** *p* < 0.001). (**C**) Particle size analysis of pure Aβ1-42 (10 μM; day 0: black line, day 5: dashed black line) and Aβ1-42 incubated with DhHP-6 (10 μM:10 μM molar ratio; day 0: red line, day 5: dashed red line) at 37 °C from day 0 to 5 by dynamic light scattering (DLS) assay. (**D**) Characterization of Aβ1-42 (10 μM) and Aβ1-42 incubated with DhHP-6 (10 μM:10 μM molar ratio) at 37 °C for three days by TEM observation, scale bar = 0.2 μm.

We designed the 1228.5 kD DhHP-6 mimetic of MP-11, with the chemical structure that is shown in Figure 1A (Figure S1). Thioflavin T (Th-T) was used to visualize stacked β sheets of amyloid fibrils in vitro and in vivo. Binding can enhance the fluorescence intensity at 450 nm (excitation wavelength) and 485 nm (emission wavelength) [35]. The increase of fluorescence intensity of Aβ1-42 was significantly reduced following incubation with DhHP-6 at a 10 μM:10 μM molar ratio (pure Aβ1-42, black line, Aβ1-42:DhHP-6, red line; Figure 1B). The DhHP-6-induced reduction in the diameter of Aβ1-42 was evaluated by dynamic light scattering (DLS). Aβ1-42 formed large aggregations with diameters ranging from 78 nm to 531 nm after five days of incubation at 37 °C. In contrast, the Aβ particles were broken down into 396-nm particles after DhHP-6 incubation for five days (Figure 1C). Furthermore, TEM showed an alteration in the supramolecular structure of Aβ1-42 (fibril formation) into uniformly dispersed spherical particles (Aβ1-42:DhHP-6 = 10 μM:10 μM molar ratio) after three days of DhHP-6 incubation at 37 °C (Figure 1D). Taken together, DhHP-6 inhibited and disassembled Aβ1-42 aggregation.

**Figure 2:**
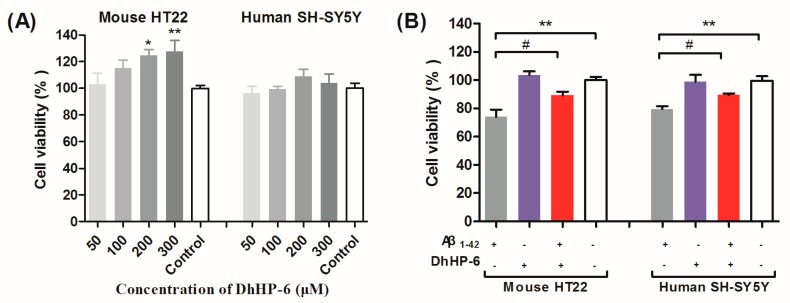
Cytotoxicity of DhHP-6 and its neuro-protective effect on Aβ1-42 treated SH-SY5Y human neuroblastoma and HT22 mouse hippocampal neuron cell lines. (**A**) DhHP-6 promoted the proliferation of HT22 cells (* *p* = 0.0226, ** *p* = 0.0036) at 200 μM and 300 μM, but had no effect on SH-SY5Y cells at 50 μM, 100 μM, 200 μM, and 300 μM. (**B**) Aβ1-42 oligomers (30 μM) showed significant cytotoxicity in both HT22 and SH-SY5Y cells, and 100-µM DhHP-6 protected cells from Aβ1-42-induced cytotoxicity (** *p* < 0.01; ^#^ *p* < 0.05).

The cytotoxic and neuroprotective effects of DhHP-6 in HT22 mouse hippocampal neuron cells and SH-SY5Y human neuroblastoma cells were also evaluated by the 3-[4,5-dimethylthiazol-2-yl]-2,5-diphenyl tetrazolium bromide (MTT) cell viability assays. DhHP-6 was non-toxic to HT22 and SH-SY5Y cells at the concentration range of 50–300 μM after 24 h of incubation (Figure 2A). Studies show that the low molecular weight Aβ1-42 oligomers are the most neurotoxic among all of the agents that cause neuronal apoptosis [13]. A high dose (100 μM) of DhHP-6 had a significant neuroprotective effect on HT22 and SH-SY5Y cells incubated with Aβ1-42 oligomers (^#^ *p* < 0.05, Figure 2B), which is likely due to the inhibition of Aβ aggregation.

### 2.2. DhHP-6 Ameliorates Cognitive Decline and Improves Hippocampus Damage in APPswe/PSEN1dE9 Mice

**Figure 3:**
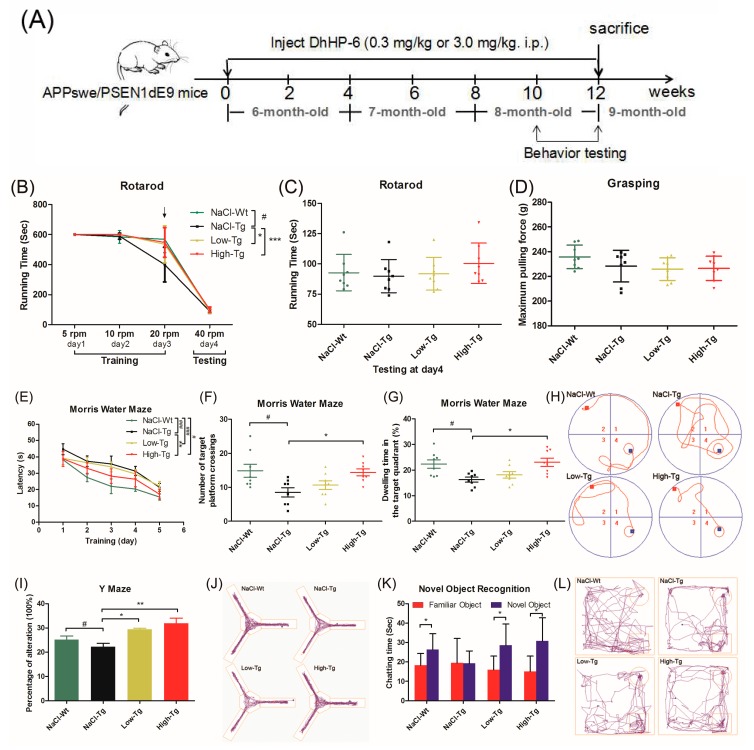

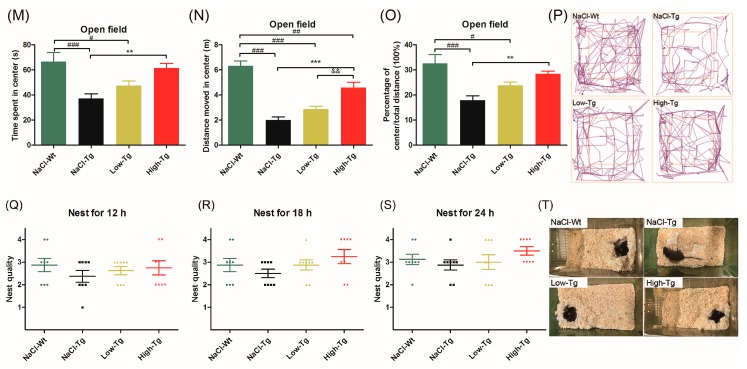
Assessment of the cognitive capacity of APPswe/PSEN1dE9 mice treated with DhHP-6. (**A**) DhHP-6 treatment strategy: APPswe/PSEN1dE9 mice received a daily intraperitoneal injection of 0.3 (Low-Tg) and 3.0 mg/kg (High-Tg) DhHP-6 for 12 weeks. Transgenic mice and wild-type mice mock-treated with NaCl were used as the control (NaCl-Tg) and negative control (NaCl-Wt) groups, respectively. (**B**) Sensorimotor tests were used to evaluate the basic behavior of mice to exclude any effect of the treatment on cognitive tests (^#^ *p* < 0.05; * *p* < 0.05; *** *p* < 0.001). (**C**) Rotarod test at 20 rpm showed a slower response of the NaCl-Tg mice compared to NaCl-Wt, Low-Tg, and High-Tg groups. (**D**) No significant difference was observed between the groups in the grasping test (D). (**E**–**H**) Morris water maze test. (**E**) Low-Tg and NaCl-Tg mice needed more time in escaping latency to find the hidden platform compared to the wild-type and High-Tg mice (^#^ *p* < 0.05; ** *p* < 0.01; ^###^ *p* < 0.001). (**F**) Total number of times the mice passed through the target platform (^#^ *p* < 0.05; * *p* < 0.05). (**G**) Time spent in the target quadrant (^#^ *p* < 0.05; * *p* < 0.05). (**H**) Typical tracks of each group. (**I**,**J**) Y-maze test. One alternation was defined as the consecutive entry of a mouse into three arms. (**I**) NaCl-Tg showed significant spatial recognition decline compared to the wild-type mice, while both Low-Tg and High-Tg mice showed improvements (^#^ *p* < 0.05; * *p* < 0.05; ** *p* < 0.01). (**J**) Typical moving tracks in different groups. (**K**,**L**) Novel object recognition test. (**K**) Low-Tg and High-Tg mice showed more preference for novel objects than for the familiar ones compared to NaCl-Tg mice (* *p* < 0.05). (**L**) Movement tracks of different groups. (**M**–**P**) Open field test. Time (^#^ *p* < 0.05; ^###^ *p* < 0.001; ** *p* < 0.01). (**M**), distance (^##^ *p* < 0.01; ^###^ *p* < 0.001; *** *p* < 0.001; ^&&^ *p* < 0.01) (**N**), relative distance (^#^ *p* < 0.05; ^###^ *p* < 0.001; ** *p* < 0.01). (**O**), and moving tracks (**P**) spent in the central area. All of the Alzheimer’s disease (AD) mice showed anxiety and less movement in exploring the central zone compared to the wild-type mice, which are effects that were improved by high-dose DhHP-6. (**Q**–**T**) Nest building. Score statistics of each group at 12 h (**Q**), 18 h (**R**), and 24 h (**S**) after building. (**T**) Typical nest results of each group.

To determine the neuroprotective effect of DnHP-6 on AD mice, we assessed the spatial learning and memory capacity on APPswe/PSEN1dE9 (APP/PS1) mice using established behavioral tests. Six-month-old male transgenic mice were divided into the following three groups (*n* = 8/group): Low Tg (treated with 0.3 mg/kg DhHP-6), High-Tg (treated with 3 mg/kg DhHP-6) and NaCl Tg (mock-treated with the same volume of saline). A negative control group comprising of wild-type male mice of the same age that were mock-treated with the same volume of saline was also included (NaCl-Wt). The mice were intraperitoneally treated with the respective reagents for 12 weeks, and the behavioral tests were started from the 10^th^ week onwards until the mice were euthanized at the end of the 12-week period (Figure 3A). Mice were weighed and subjected to visual tests before the behavioral tests to exclude the treatment effects and individual differences [23,36]. General motor functions, coordination, and balance were evaluated by the rotating bar and grasping tests. Low-Tg, High-Tg and NaCl-Wt groups displayed better endurance and reflexes at a high rotational speed (20 rpm) compared to the NaCl-Tg mice during the rotating bar training period (Figure 3B), while all of the mice showed similar rotarod grasping abilities with no observable anomalies during the testing period (Figure 3C,D). This result indicated that DhHP-6 had no adverse effects on mouse behavior. In the Morris water maze test, all of the transgenic mice (Low-Tg, High-Tg, and NaCl-Tg) needed more time to escape latency and find the hidden platform compared to the wild-type mice (NaCl-Tg) during the pre-training test (^###^ *p* < 0.001, ^#^ *p* < 0.05 and ^###^ *p* <0.001). Compared to NaCl-Tg, the High-Tg group showed significantly shorter duration of escaping latency (** *p* < 0.01; Figure 3E), indicating cognitive improvement. After removing the hidden platform, the NaCl-Tg mice had greater difficulty finding the previous location of the platform site compared to the NaCl-Wt mice (^#^ *p* < 0.05), indicating a significant spatial memory disorder. The High-Tg mice spent more time in the platform quadrant and passed over the platform site more times compared to the NaCl-Tg mice (* *p* < 0.05; Figure 3F,G), indicating that DhHP-6 restored the cognitive decline in AD mice. The typical swimming tracks of each group (Figure 3H) also showed that mice treated with DhHP-6 had a significant improvement in spatial memory.

Spatial memory loss caused by amyloid deposition was also evaluated by the Y-maze test [37]. Both Low-Tg and High-Tg mice showed significantly better spatial recognition ability than the NaCl-Tg group (* *p*< 0.05 and ** *p* < 0.01) in terms of entering into the three arms consecutively (Figure 3I,J). Furthermore, short-term memory and learning, and the preference for novelty, was evaluated by novel object recognition (NOR) assay. APP/PS1 mice treated with DhHP-6 showed better social interaction compared to NaCl-Tg mice in terms of preference for a novel object as opposed to a familiar one (Figure 3K,L; * *p* < 0.05). The exploration and anxiety-related behaviors were evaluated using the open field test [38], and NaCl-Tg mice showed lower exploration ability compared to NaCl-Wt, while the High-Tg mice paid more attention to exploring the middle space (Figure 3M–P; ^###^ *p* < 0.001, ** *p* < 0.01 and *** *p* < 0.01).

Non-maternal nest building performance is sensitive to hippocampal damage and used to evaluate the murine models of psychiatric disorders [39,40]. During 12 h of nest-building, the average score of nesting was two (Figure 3Q), which improved in the DhHP-6-treated groups at 18 h (Figure 3R). High-Tg group mice could build almost perfect nests at 24 h (Figure 3S). Representative results of the nest-building test are shown in Figure 3T. Therefore, the DhHP-6-treated AD mice had better nest-building capacity over time compared to the NaCl-Tg mice. All of the above behavioral tests indicated that DhHP-6 ameliorated hippocampal damage-related spatial memory and cognitive decline, enhanced short-term memory-related spatial exploration ability and novel object recognition capacity, reduced anxiety, and improved nest-building performance in APP/PS1 mice.

### 2.3. DhHP-6 Significantly Reduces Amyloid Deposition and Reverses Neuronal Atrophy in the Cortex and Hippocampus of APPswe/PSEN1dE9 Mice

**Figure 4:**
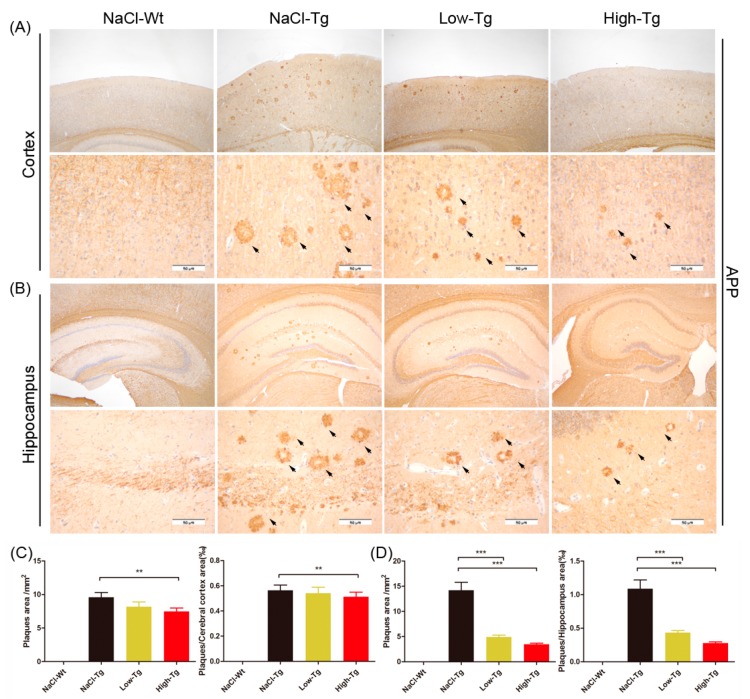
Immunohistochemical (IHC) analysis of amyloid plaque deposits in the cortical and hippocampal section of APPswe/PSEN1dE9 mice treated with DhHP-6. (**A**,**B**) Representative IHC photomicrographs of amyloid plaques (at the arrowhead) in the cerebral cortex and hippocampus of APPswe/PSEN1dE9 mice, scale bar = 50 μm. Amyloid plaque area in the cerebral cortex (** *p* < 0.01, **C**) and hippocampus (*** *p* < 0.001, **D**) of APP/PS1 mice.

Amyloid deposition in the brain of both AD patients and animal models is an important biomarker of AD pathogenesis and treatment response [41]. Since cognitive improvement is associated with a reduction in amyloid deposition in both the cerebral cortex and hippocampus, we evaluated Aβ peptides and APP in the wild-type and APP/PS1 mice. Compared to the NaCl-Tg group, the Low-Tg and High-Tg mice had fewer and smaller amyloid plaques in both the cerebral cortex (Figure 4A,C) and hippocampus (Figure 4B,D). The number of plaques and plaque area in the cerebral cortex was reduced by 4.1% and 9% respectively in the Low-Tg mice, and by 14.9% and 22.2% respectively in the High-Tg mice compared to the NaCl-Tg group (Figure 4C), and those in the hippocampus were reduced by 60% and 74.4%, and by 65.4% and 73.6% in the Low-Tg and High-Tg mice, respectively (Figure 4D). Therefore, DhHP-6 remarkably reduced the amyloid burden in both the cerebral cortex and hippocampus. 

**Figure 5:**
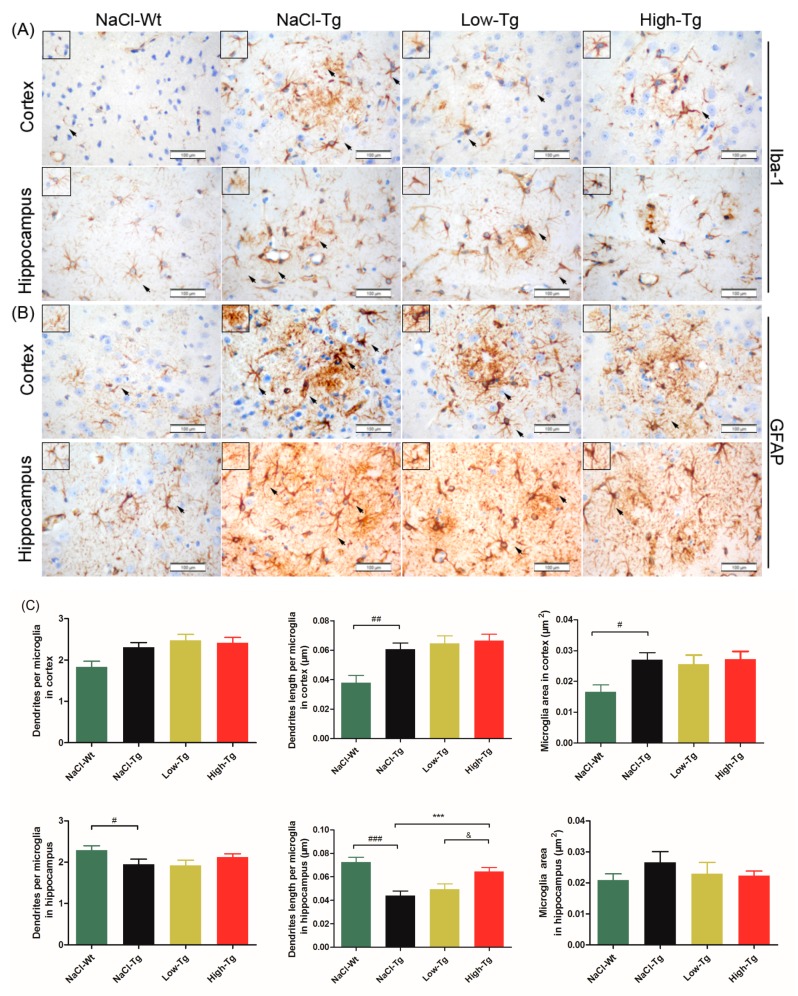

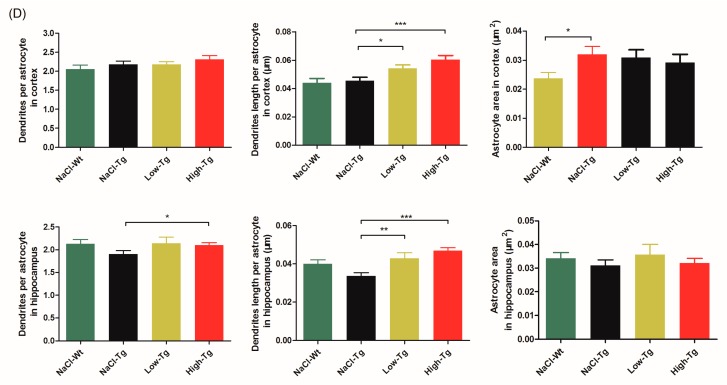
Immunohistochemical (IHC) analysis of astrocytes and microglia in the cortical and hippocampal section of APPswe/PSEN1dE9 mice treated with DhHP-6. (**A**,**B**) Representative IHC photomicrographs of astrocytes (GFAP+) and microglia (Iba-1+) in the cerebral cortex and hippocampus of APPswe/PSEN1dE9 mice, scale bar = 100 μm (insert graph and arrows: the typical morphological characteristics of neurons). (**C**) Number of dendrites, dendritic length, and microglial area. The number of dendrites around the neurons was defined as dendrites (^#^ *p* < 0.05; ^##^ *p* < 0.01; ^###^ *p* < 0.001; *** *p* < 0.001; ^&^ *p* < 0.05). (**D**) Astrocyte score and morphology (* *p* < 0.05; ** *p* < 0.01; *** *p* < 0.001).

Aβ deposition exacerbates neuroinflammation, resulting in neuronal atrophy. The clustering of microglia and astrocytes around Aβ plaques is a pathological hallmark of AD [42]. Activated microglia and astrocytes in the cerebral cortex and hippocampus were observed by targeting Ionized calcium-binding adaptor molecule 1 (Iba-1, Figure 5A,C) and Glial fibrillary acidic protein (GFAP, Figure 5B,D), respectively. Microglia are the primary immune cells of the central nervous system (CNS), and often surround the plaque deposits, increasing in number with the plaque size [43]. Iba-1+ microglia were observed in the cerebral cortex and hippocampus of all of the mice, while only few and well-distributed microglia with normal morphology were seen in NaCl-Wt; numerous amoeba-like microglia were present around the Aβ plaques in the NaCl-Tg mice. DhHP-6 treatment partially restored the morphological damage and atrophy of microglial cells (Figure 5A). In addition, the hippocampal microglia of NaCl-Tg mice had fewer and shorter dendrites compared to NaCl-Wt mice (^#^ *p* < 0.05 and ^###^ *p* < 0.001), and DhHP-6 at high doses significantly restored the length of dendrites (*** *p* < 0.001). 

Astrocytes are abundant and heterogeneous glial cells that accumulate extensively at sites of Aβ deposition [44]. A dense mass of GFAP+ cells with apparent atrophy, smaller cell bodies, and fewer and shorter processes were seen around the Aβ plaques of NaCl-Tg mice. In contrast, the astrocytes of NaCl-Wt mice had normal morphology, dispersed uniformity, and longer processes. The active astrocytes in the Low-Tg and High-Tg mice showed improvement with more and longer processes compared to the NaCl-Tg mice, both in the cortex (length of dendrites in Low-Tg, * *p* < 0.05 and High-Tg, *** *p* < 0.001) and hippocampus (length in Low-Tg, ** *p*< 0.01, and High-Tg, *** *p* < 0.001) (Figure 5D). Taken together, these results showed that DhHP-6 alleviated brain inflammation and reversed neuronal atrophy in the cortex and hippocampus of APPswe/PSEN1dE9 mice. 

### 2.4. Pro-Inflammatory Cytokine Levels in DhHP-6 Treated AD Mice

**Figure 6:**
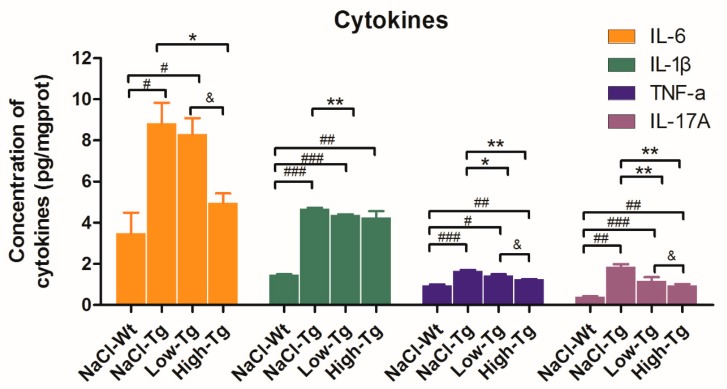
Pro-inflammatory cytokine level in APPswe/PSEN1dE9 mice treated with DhHP-6. Pro-inflammatory cytokines IL-6, IL-1β, IL-17A, and TNF-α were significantly higher in the brain of AD mice compared to the wild-type mice, and were significantly downregulated after DhHP-6 treatment (^#^ *p* < 0.05; ^##^ *p* < 0.01; ^###^ *p* < 0.001; * *p* < 0.05; ** *p* < 0.01; ^&^ *p* < 0.05).

Aβ deposition in the brain induces an inflammatory response via the activation of microglia and astrocytes, which produce pro-inflammatory factors such as tumor necrosis factor-α (TNF-α), interleukin-1β (IL-1β), or IL-6, and growth factors and extracellular matrix proteins [45]. Therefore, the level of these cytokines can reflect the extent of Aβ deposition, AD progression, and treatment efficacy. AD transgenic mice had significantly higher levels of IL-6, IL-1β, TNF-α, and IL-17A compared to wild-type mice, as indicated by ELISA. In addition, a significant decrease was seen in the IL-6 levels in the High-Tg mice and the IL-1β levels in the Low-Tg mice. There was an overall significant reduction in the level of IL-17A and TNF-α in the DhHP-6-treated groups compared to the untreated group, indicating that DhHP-6 mediated its effect by immunomodulatory pathways (Figure 6).

## 3. Discussion

AD is a common progressive neurodegenerative disorder characterized by cognitive decline, memory impairment, and behavioral anomalies. Several hypotheses have been proposed to explain the molecular and pathological mechanisms of AD, and among them, the amyloid cascade hypothesis is the most widely accepted. The extracellular aggregation of the insoluble proteinaceous amyloid β plaques and/or the soluble toxic oligomers of Aβ1-42 exacerbate oxidative stress and trigger mitochondrial dysfunction, leading to neuronal death [46]. Increased ROS levels further aggravate the β and γ-secretase cleavage of APP and result in the production of more Aβ [10]. Over time, these pathomolecular changes in the intracerebral tissues result in psychological and metabolic disorders. Compelling evidence shows that reducing the production and formation of Aβ plaques and regulating the redox homeostasis are effective therapeutic strategies for AD [47,48,49]. McKoy et al. [46] screened 65,000 small molecules and found that D737 inhibited Aβ1-42 aggregation, reduced Aβ1-42-induced cellular dysfunction and ROS production, and prolonged the lifespan and improved the locomotive ability of a Drosophila melanogaster model of AD. Sharma and Paul [50] showed that caffeine inhibited the aggregation of Aβ16-22 peptide by stimulating its molecular dynamics in a transgenic mouse model of AD. Zhang et al. [51] reported on the antioxidant and neuroprotective effects of a natural bioactive tetrapeptide (TPM) derived from maize in a *C. elegans* model of AD (strain GM101). It inhibits Aβ aggregation, scavenges ROS, reduces Aβ, and maintains full-length Aβ1-42 expression in the nematode, thereby increasing the lifespan, and abrogating paralysis and plaque deposition. 

DhHP-6 is a mimetic of the natural MP-11 with a high peroxidase activity of 3.9 × 10^3^ U/μM (i.e., 93% of that of MP-11) that was firstly designed to treat cataracts [32]. DhHP-6 also extends the lifespan of *C. elegans* and retards the aging process by enhancing the activity of the upstream transcriptional regulator DAF-16 [34], which activates stress-responsive, antimicrobial, and metabolic genes including superoxide dismutase (*sod-3*), transmembrane tyrosine kinase (*old-1*), metallothioneine (*mtl-1*), and small heat-shock proteins [52,53]. On the basis of these findings, DhHP-6 was first tested in the transgenic *C. elegans* strain CL4176 expressing Aβ1-42, and resulted in improved survival, and a reduction in paralysis and plaque deposition (data not shown). To determine the underlying mechanism of the potential neuroprotective effect of DhHP-6 in the mammalian system, we used the APPswe/PSEN1dE9 transgenic mouse model of AD. 

The Aβ aggregates are typically characterized by the highly ordered β-sheet conformation [54]. DhHP-6 treatment significantly decreased the fluorescence intensity of Thioflavin T binding with β-sheets, indicating that it inhibited the accumulation and aggregation of Aβ peptides. In addition, Aβ1-42 incubated with DhHP-6 had a smaller particle size and a uniform size distribution, as evaluated by the DLS test and TEM, indicating the direct interaction of DhHP-6 with Aβ, which prevented the latter’s aggregation. This is clinically relevant, since Aβ-induced neurotoxicity occurs in the early stages of fibril formation. Finally, DhHP-6 is non-toxic to both the murine hippocampal HT22 and human SH-SY5Y neuroblastoma cells.

APPSwe/PS1dE9 (APP/PS1) transgenic mice overexpressing the human mutated APP with Swedish mutation (APP695Swe), and mutant human presenilin 1 with exon 9 deletion (PSEN1dE9), are characterized by age-related memory deficit, anxiety, hyperactivity, and impaired social interaction [55]. APP/PS1 mice are widely used as an AD model due to progressive age-related Aβ accumulation, cerebral amyloidosis and associated pathologies, and cognitive deficits [56]. We treated six-month-old APP/PS1 transgenic male mice with either 0.3 mg/kg (Low-Tg) or 3.0 mg/kg (High-Tg) of DhHP-6, or with the equivalent volume of 0.9% saline (NaCl-Tg) for 12 weeks, and we included a wild-type, saline-treated negative control group (NaCl-Wt). NaCl mice were slower compared to the mice of the other groups in responding to the sensorimotor rotating bar and grasping tests, while the other mice showed no significant differences in physical capacity, grasping, and vision. In the Morris water maze experiment, Low-Tg and High-Tg mice performed better in terms of escaping latency and locating the hidden platform compared to the NaCl-Tg mice, and were similar to the NaCl-Wt mice. After removing the platform, DhHP-6-treated mice were able to concentrate on searching for the previous location of the platform, and although they spent more time on passing repeatedly through the quadrant compared to the wild-type mice, they performed significantly better than the NaCl-Tg mice. Therefore, DhHP-6 treatment improved the spatial learning ability and rescued memory loss in AD mice. Similar results were obtained by Y-maze, novel object recognition, and open field tests, with the DhHP-6-treated mice showing significantly higher spatial recognition and memory abilities in terms of the alternation ratio and preference for novel objects, as well as better exploratory and lower anxiety than the NaCl-Tg mice. Furthermore, the DhHP-6 treated mice had higher nest-building scores and improved nest-building capacity with time, indicating the restoration of the hippocampal function. Taken together, DhHP-6 resulted in a significant improvement in short-term memory, cognitive capacity, and AD-related pathology.

The deposition of Aβ1-42 positive plaques in the cerebral cortex and hippocampus is the strongest evidence of AD, and is used to monitor the therapeutic response [57]. We observed a significant reduction in plaque density in DhHP-6-treated mice compared to the untreated mice, as well as in the Aβ1-42 aggregates in the cellular AD model. Furthermore, as regards the microglial cell morphology and distribution, the Iba-1+ microgliosis area and GFAP+ astrogliosis area were significantly restored in the DhHP-6-treated AD mice. In addition, DhHP-6 reduced the level of pro-inflammatory cytokines such as IL-6, IL-1β, IL-17A, and TNF-α, indicating the important role of the mimetic peptide in regulating the inflammatory and immune response. Taken together, DhHP-6 inhibited Aβ aggregation in vitro and amyloid plaque formation in vivo, showed significant neuroprotective effects, and improved the cognitive and memory defects in the AD mouse model, probably by regulating the immune response. Nevertheless, further studies are needed to evaluate the underlying mechanisms of DhHP-6-mediated free radical scavenging, the inhibition of Aβ aggregation, and immunomodulation. In a recent study of our group, molecular docking analysis showed that the histidine2-iron-porphyrin complex of the DhHP-6 active site could bind closely with the β-sheets of the Aβ peptides. This indicates that as a peroxidase mimetic, DhHP-6 might have bifunctional antioxidant and Aβ disassembly properties. The interaction between DhHP-6, Aβ, the antioxidant-related indices, and potential targets should be further studied. 

At present, amyloid cascade targeting drugs such as aducanumab (BIIB037) [58], ALZT-OP1, and GV-971 [24] are still in phase III clinical trials. Our current study provides further experimental evidences of the potential therapeutic role of DhHP-6 in AD.

## 4. Materials and Methods

### 4.1. Materials and Regents 

DhHP-6 was designed and synthetized in our laboratory on solid phase, as previously described [31]. Thioflavin T (Th-T) and hexafluoroisopropanol (HFIP) were purchased from Sigma-Aldrich (St Louis, MO, USA). The SH-SY5Y human neuroblastoma cell line and HT22 mouse hippocampal neuron cell line were purchased from the American Type Culture Collection (ATCC, Manassas, VA, USA). Aβ1-42 peptide and cell culture reagents were of analytical grade and purchased from Meilunbio Co., Ltd. (Dalian, China).

### 4.2. Design and Synthesis of Peroxidase Mimetic

Peroxidase mimetic deuterohemin-AlaHisThrValGluLys (DhHP-6) was synthesized on rink-amide resins by solid-phase peptide synthesis. Crude peptides were obtained by cleavage solution (trifluoroacetic acid:triisopropylsilane:water = 95:2.5:2.5) followed a standard protocol, purified by high-performance liquid chromatography System (HPLC, Agilent Technologies, Santa Clara, CA, USA) equipped with a C18 column, and characterized by MALDI-TOF/TOF mass spectrometer (AB SCIEX, Framingham, MA, USA).

### 4.3. Measurement of Aβ Fibrils by Thioflavin T Assay

Aβ1-42 peptide (10 μM) and DhHP-6 incubated with Aβ1-42 (10:10 molar mass ratio) were dissolved in phosphate buffer (PB; pH 7.4) with thioflavin T (Th-T) at 37 °C. Fluorescence intensity was measured using a microplate reader (GENIOS, Tecan, US) at various incubation times and the 450-nm excitation wavelength according to a standard thioflavin T assay protocol [59].

### 4.4. Dynamic Light Scattering (DLS)

Pure Aβ and Aβ mixed with DhHP-6 (10 μM:10 μM molar ratio) were incubated at 37 °C for five days. All of the samples were dispersed in milliQ water and then transferred to a cuvette, and the hydrodynamic particle size was analyzed at 25 °C by dynamic light scattering (DLS) technique using a Malvern Zeta sizer Nano ZS Instrument NANO ZS90 (Malvern Instruments Ltd., Worcestershire, UK) [60,61].

### 4.5. Transmission Electron Microscopy (TEM)

Pure Aβ and Aβ mixed with DhHP-6 (10 μM:10 μM molar ratio) were dissolved in phosphate buffer (pH 7.4) and incubated at 37 °C for three days. Then, an aliquot of 10 μL from each sample was placed onto TEM grids, dried, and viewed using a JEM-2100F microscope with a field emission gun operating at 200 kV. 

### 4.6. Cell Viability Assay 

SH-SY5Y and HT22 cells were maintained in DMEM (supplemented with 10% FBS (the fetal bovine serum), 100 units penicillin and 100 µg/mL streptomycin) and cultured at 37 °C with 5% CO_2_. Aβ1-42 oligomer (AβO) was prepared as previously described [62]. Pure Aβ1-42 peptides were dissolved in hexafluoroisopropanol (HFIP) and volatilized to form a peptide membrane, followed by dissolution in 20 µL of DMSO. Ice-cold phenol-free Ham’s F-12 cell culture medium was added, and the sample was incubated at 4 °C for 24 h to obtain a 1 mM of AβO stock solution. DhHP-6 was dissolved in DMEM basic medium to obtain a 500-µM DhHP-6 stock solution.

As regards the cytotoxicity test, SH-SY5Y and HT22 cells (2 × 10^5^ cells/mL) were seeded in a 96-well cell culture plate at 100 μL/well and cultured for 12 h; then, they were exposed to 50–300 µM of DhHP-6 for 24 h. As regards the neuroprotective test, cells were pre-treated with DhHP-6 (100 µM) for four hours, and then treated with AβO (30 μM) for 24 h. Subsequently, MTT solution (5 mg/mL, 20 μL/well) was added, and cells were incubated for 4 h at 37 °C. Next, MTT was removed, DMSO (150 μL/well) was added, and the absorbance was read at 490 nm by an ELISA reader (Bio-Rad, Hercules, CA, USA) [60].

### 4.7. Transgenic Mice and Treatment

Two-month-old male transgenic mice, B6C3-Tg (APPswe/PSEN1dE9)/Nju (APP/PS1) and B6C3F1-Wt (Wild-type), were provided by the Model Animal Research Center of Nanjing University in China. All of the animals were housed in standard cages under 12 h/12 h light/dark cycles, with free access to food and water. The animal experimental procedure was approved by the Animal Care Committee of Jilin University (License No.: 20160518) at 18 May 2016 and conformed to the Animal Ethical Standards and Use Committee at Jilin University [63].

Six-month-old male transgenic mice were divided into the following three groups (n = eight/group): Low-Tg (treated with 0.3 mg/kg DhHP-6), High-Tg (treated with 3 mg/kg DhHP-6), and NaCl-Tg (mock treated with the same volume of saline). Wild-type male mice of the same age were treated with the same volume of saline as the negative control (NaCl-Wt). Mice were intraperitoneally treated with the respective reagents for 12 weeks, and the behavioral tests were started from the 10th week onwards until the mice were euthanized at the end of the 12-week period. All of the tests were conducted by an individual who was blind to the treatment assignment. 

Mice were weighed and subjected to visual tests before the behavioral tests to exclude treatment effects and individual differences. The sensorimotor tests, including the rotating bar and grasping tests, were used to measure general motor functions, coordination, and balance. The behavioral testing, including Morris water maze, Y-maze, novel object recognition, open-field, and nest-building tests, were used to assess short-term memory as well as working memory, spatial memory ability, anxiety, social interaction capacity, and hippocampus damage relative cognitive behavioral changes. All of the instruments were purchased from ZS Dichuang, China. Mice movement tracking was recorded by an automated video tracking system (San Diego Instruments, San Diego, CA, USA).

#### 4.7.1. Rotary Rod

Mice were placed on a 30-mm diameter rod of the rotaryrod fatigue tester to keep the balance for 60 s, and trained for 10 min under a speed of five rpm on the first training day, 10 rpm on the second training day, and 20 rpm on the third training day. Then, on day four (the testing day), the speed of the rod was set up to 40 rpm. Mice falling time and rod speed were recorded (within 10 min) when the mice fell from the top of the rotary rod. Skimmed cotton was placed under the rod to prevent injury from falling.

#### 4.7.2. Grasping

Limb strength and body movement-related neural lesion were measured by the grasping test. During the test, mice were placed on the surface of a 230 mm × 250 mm grasping test plate. The grasping force was tested by the sustained and stable tension of each mouse for three trials. The maximum pulling force was considered as the grasping mice force.

#### 4.7.3. Morris Water Maze

The Morris water maze, as the typical assessment of cognitive capacity, was used for evaluating learning and memory ability [64]. The water maze apparatus was built in a white iron pool (120-cm diameter, 40-cm high) with a fixed white circular platform (80-mm diameter) hidden 1–2 cm below the surface of the water (22 ± 1 °C). For the acquisition phase, mice were placed into each quadrant of the four, dividing the pool (north, south, east, and west, in turn) to find the platform that was hidden under the water at a fixed position for five days of daily trials. Mice that escaped the latency time to find the platform were recorded (within 60 s), or the mice were guided to the platform by the experimenter, and the time was recorded as 60 s. On day six, the platform was removed from the pool, and mice were allowed to explore the pool for 60 s. During the testing phase, the escaping latency time, the number of mice crossing over the target platform (where the platform was located during the hidden platform training), the time spent in the target quadrant, and the movement tracking were recorded by an automated video tracking system.

#### 4.7.4. Y-Maze

The Y-maze apparatus consisted of three radial 30-cm long arms (named as starting, novel, and other arms) originating from the central space to form a “Y” shape. Mice were placed into the starting arm to explore the maze based on the rodent’s innate curiosity to explore novel areas. Briefly, mice were placed into starting arm to explore and locate the novel arms freely by spatial clues for 10 min (training period). After a 2-h interval, mice were placed into the Y-maze again as part of the training period protocol to evaluate spatial memory. Time, distance, enter times, and movement tracks were recorded by an automated video tracking system.

#### 4.7.5. Novel Object Recognition (NOR)

Novel object recognition is widely used to measure short-term memory and learning, the preference for novelty, and the influence of the hippocampus area in the process of recognition in rodents [65]. It was performed in a square-shaped open-field box (300 mm square × 300 mm high walls made of black Plexiglas with a white floor) with objects located at the opposite site of the starting point. Briefly, mice were allowed to explore two identical objects (cylinder) in the open field for 10 min (learning period). After a 24-h interval, mice were allowed to explore the one familiar object (cylinder) and one novel object (cuboid) as part of the learning period protocol. The time spent exploring familiar and novel objects, and the movement tracks of mice, were recorded by a tracking system [23,66].

#### 4.7.6. Open Field

The open field test measures the exploration of a new environment and anxious behavior, which is based on the idea that mice naturally prefer to be near a protective wall rather than exposed to danger out in an open field [37,67]. It was performed in a square-shaped open-field box (as the open-field apparatus described above). The zone could be set into an inside square (150 mm) as the “center area” and an outside square as the “surrounding area”. Each mouse was gently placed on the floor and allowed to freely explore the area for 10 min to investigate the spontaneous locomotor activity. Their overall time, distance, and movement tracks in the center and surrounding areas were measured by a tracking system. 

#### 4.7.7. Nest-Building Test

The nest-building test was used to detect hippocampus damage and evaluate murine models of psychiatric disorders [39]. Each mouse was housed in a single cage before testing. During the nest-building test, clear sawdust and two pieces of compressed cotton (5 cm × 5 cm) were placed inside the cage for nesting. A peaceful and quiet environment was needed for nest-building. The presence and quality of the nest were scored into four grades: score 1: not noticeably touched; score 2: partially torn up; score 3: mostly shredded but flat; and score 4: perfect or nearly perfect. All of the nests were scored at 12 h, 18 h, and 24 h during the nest-building period. 

### 4.8. Immunohistochemistry 

After mice sacrifice, brains were excised, fixed in 4% paraformaldehyde, and embedded in paraffin. Serial coronal brain sections were cut along the entire rostro-caudal brain axis and stained for IHC analysis using a standard protocol [63]. After blocking with 5% normal goat serum, the section was incubated with anti-amyloid precursor protein monoclonal antibody (1:500, Abcam, Cambridge, UK), anti-Iba1 monoclonal antibody (1:40, Abcam, Cambridge, UK), and anti-GFAP monoclonal antibody (1:300, CST, Massachusetts, USA) at 4 °C overnight. Sections were then washed with PBS, incubated with horseradish peroxidase (HRP)-labeled sheep anti-mouse/rabbit secondary antibody, and then with the chromogen diaminobenzidine (DAB).

### 4.9. Inflammatory Cytokines

After mice sacrifice, brains were excised and immediately frozen in liquid nitrogen. Then, the brain samples were homogenized in 0.9% normal saline containing a protease inhibitor cocktail (Meilunbio Co., Ltd., Dalian, China), followed by ultrasonication for 10 min. After centrifugation, the supernatant was collected to measure cytokine levels using mice IL-1β, IL-6, IL-17A, TNF-α quantikine ELISA kits (R&D systems) according to the manufacturer’s instructions.

### 4.10. Statistical Analysis

Image-Pro Plus 6.0 software (Media cybernetics, Rockville, MD, USA) was used to analyze plaques, GFAP (astrocytes biomarker), and Iba-1 (microglia biomarker) in the cerebral cortex and hippocampus. Statistical analysis was carried out using the *t*-test for two independent samples, and ANOVA analyses of Tukey’s multiple comparison test for studies comparing four groups in behavioral tests. All of the statistical analyses were performed using GraphPad Prism 5 (GraphPad Software, La Jolla, CA, USA) for testing the significance of the results. Each experiment was performed in triplicate; data are presented as mean ± SD; and a *p* value < 0.05 was considered statistically significant.

## 5. Conclusions

DhHP-6 significantly reduced Aβ aggregation and size in vitro, and ameliorated the cognitive decline and hippocampal damage in a murine AD model by inhibiting the deposition of Aβ plaques. Mechanistically, DhHP-6 restored the morphology of astrocytes and microglia, and significantly reduced the levels of pro-inflammatory cytokines. Therefore, DhHP-6 is a promising therapeutic candidate for AD. Moreover, recently studies proposed Aβ could reduce sirtuin 1 and its downstream signaling, resulting in increased intracellular ROS accumulation and mitochondrial dysfunction. It is worthwhile to conduct further studies and tests.

## Supplementary Materials

The following are available online at http://www.mdpi.com/1422-0067/19/11/3304/s1, Figure S1: The High Performance Liquid Chromatography (A) and Time of Flight Mass Spectrometer (B) of the DhHP-6 (M = 1228.7524 ± 1).

## Abbreviations

AD	Alzheimer’s disease
Aβ	amyloid β
DhHP-6	Deuterohemin-AlaHisThrValGluLys
MP-11	microperoxidase-11
*C. elegans*	*Caenorhabditis elegans*
APP	amyloid precursor protein
ROS	reactive oxygen species
BBB	blood-brain barrier
ELISA	enzyme-linked immunosorbent assay
Th-T	Thioflavin T
HFIP	hexafluoroisopropanol
AβO	Aβ1-42 oligomers
NOR	Novel Object Recognition
DLS	Dynamic light scattering
ANOVA	one-way analysis of variance
