# Supplementary material for: A Novel Peroxidase Mimics and Ameliorates Alzheimer’s Disease-Related Pathology and Cognitive Decline in Mice
Source: Int J Mol Sci. 2018 Oct 24;19(11):3304. doi: 10.3390/ijms19113304 (PMC6274722; doi:10.3390/ijms19113304)
Supplement: Supplementary file 1 [file ijms-19-03304-s001.pdf]

A Novel Peroxidase Mimics Ameliorates Alzheimer's Disease-related Pathology and Cognitive Decline in mice

Jia Xu, Kai Wang, Ye Yuan, Hui Li, Ruining Zhang, Shuwen Guan, Liping Wang

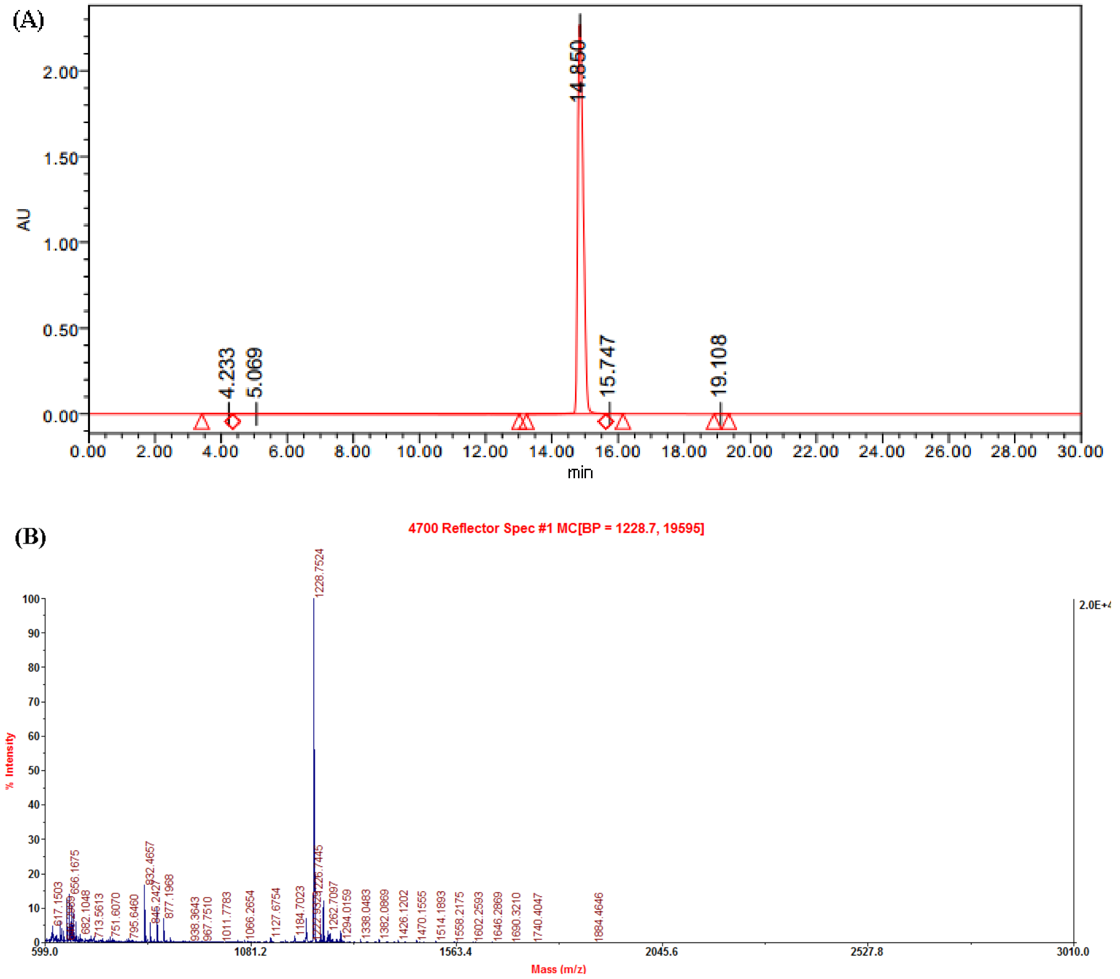

Figure S1. The High Performance Liquid Chromatography (A) and Time of Flight Mass Spectrometer (B) of the DhHP-6 (M=1228.7524±1).
